# The value of concentration of alveolar nitric oxide in diagnosing small airway dysfunction in patients with stable asthma

**DOI:** 10.1111/crj.13565

**Published:** 2022-12-12

**Authors:** Jing Wang, Ke Wu, Xianliang Cheng, Xiangsong Chen, Yanan Qi, Limin Zhao

**Affiliations:** ^1^ Department of Infectious and Respiratory Critical Care Medicine Xinxiang Central Hospital, The Fourth Clinical College of Xinxiang Medical University Xinxiang China; ^2^ School of Clinical Medicine, Guizhou Medical University Guiyang China; ^3^ Department of Respiratory and Critical Care Medicine Huazhong Fuwai Cardiovascular Disease Hospital Zhengzhou China; ^4^ Department of Pulmonary and Critical Care Medicine Henan Provincial People’s Hospital; People’s Hospital of Zhengzhou University; People’s Hospital of Henan University Zhengzhou 450003 China

**Keywords:** asthma, CANO, lung function, small airway function

## Abstract

**Background:**

Exhaled nitric oxide (FeNO) is a simple, noninvasive, and reproducible test, and FeNO (50 ml/s) is often used to reflect airway inflammation. The peripheral small airway/alveolar nitric oxide (NO) concentration is derived from the output of NO at multiple flow rates. Concentration of alveolar NO (CANO), which has been reported to reflect peripheral small airway inflammation, may be related to parameters that reflect abnormal small airway function.

**Aim:**

This study aims to investigate the relationship among CANO levels, clinical features, and small airway function‐related indicators in patients with stable asthma and to provide a simple method for monitoring small airway function in asthma.

**Design and Methods:**

We recruited 144 patients with well‐controlled, stable asthma, including 69 patients with normal small airway function (normal group) and 75 patients with small airway dysfunction (abnormal group). CANO and pulmonary function were measured.

**Results:**

CANO was significantly higher in the abnormal group ([7.28 ± 3.25] ppb) than the normal group CANO ([2.87 ± 1.50] ppb). FEF25–75%pred ([55.0 ± 16.5]%), FEF50%pred ([46.4 ± 13.2]%), and FEF75%pred ([41.9 ± 13.1]%) in abnormal group were significantly lower compared with normal group ([89.9 ± 7.5]%), ([80.9 ± 6.8]%), and ([73.8 ± 5.0]%). CANO was negatively correlated and FEF25–75%pred, FEF50%pred, and FEF75%pred (*r* = −0.87, *P* < 0.001; *r* = −0.82, *P* < 0.001; *r* = −0.78, *P* < 0.001). CANO was positively correlated with age (*r* = 0.27, *P* = 0.001). The area under the ROC curve was 0.875 for CANO. The optimal cutoff point of 5.3 ppb had sensitivity and specificity values of 72% and 92% in diagnosing small airway dysfunction.

**Conclusion:**

CANO has diagnostic value for small airway dysfunction, and the optimal cutoff value is 5.3 ppb. However, the diagnostic evidence is still insufficient, so it still needs further exploration for its value in detecting small airway dysfunction.

## Graphical abstract

CANO may be a simpler and more convenient indicator for assisting the diagnosis of small airway dysfunction in stable asthma.

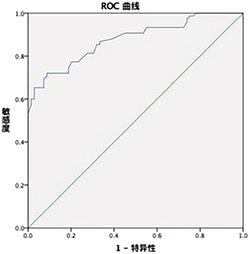

## 1 INTRODUCTION

Airway inflammation is one of the main features of bronchial asthma. It is the basis of airway hyperresponsiveness and airway remodeling, involving large and small airways and alveoli, resulting in varying degrees of airway hyperresponsiveness and bronchoconstriction. Some studies using excised lung tissue and transbronchial biopsy specimens have shown that inflammation and structural changes in the small airways and alveoli are comparable to those in the central airway.^1^, ^2^, ^3^ In order to assess peripheral small airway inflammation, transbronchial biopsy can be used for research purposes, but the invasiveness limits its routine clinical application. Although high‐resolution CT has been used to assess abnormal changes of peripheral small airway, the increased exposure to radiation caused by repeated examinations also limits its clinical application. Therefore, a noninvasive method is needed to assess peripheral small airway and optimize the daily management of asthma.

Exhaled nitric oxide (FeNO), as a noninvasive, simple and repeatable test, has been widely used in the monitoring of asthma patients. FeNO (measured at the flow of 50 ml/s) is often used to reflect airway inflammation. According to the FeNO at different flow rates (50 and 200 ml/s), concentration of alveolar nitric oxide (NO) (CANO) is calculated through two‐compartment model.^4^ And CANO is often a sensitive indicator of inflammation in the peripheral airway.^5^ The correlation between CANO levels and eosinophil counts in bronchoalveolar lavage in past literature has been confirmed, suggesting that CANO can reflect peripheral small airway/alveolar inflammation. Sustained peripheral small airway inflammation may cause small airway injury. Therefore, CANO reflecting peripheral small airway inflammation may be associated with parameters reflecting small airway dysfunction, such as FEF25–75 and FEF50. The published evidence to date has shown that the forced oscillation technique (FOT) is a noninvasive method to assess airway function by emitting oscillatory signals into the respiratory tract during tidal ventilation.^6^ In this study, the relationship between CANO levels and clinical features and small airway function indicators in patients with stable asthma was observed as follows.

## 2 MATERIALS AND METHODS

### 2.1 Subjects

A retrospective analysis was made of 144 well‐controlled asthmatic patients who visited the respiratory department of Henan Provincial People's Hospital from January 2017 to January 2018. This study enrolled these patients according to the following criteria: (1) Patients were diagnosed according to Global Strategy for Asthma Management and Prevention 2015^7^; (2) all patients were stable and had no signs of limited activity or exacerbations; (3) each patient had been treated with ICS or ICS combined with other medications for controlling asthmatic symptoms; (4) age >18 years old; and (5) CANO was performed on the same day as pulmonary function tests. Exclusion criteria are as follows: (1) oral or intravenous use of corticosteroid in the first 4 weeks; (2) complicated with obstructive or restrictive ventilatory dysfunction; (3) abnormal chest imaging findings; and (4) other serious diseases. The basic information of patients was recorded, including sex, age, height, weight, and asthma treatment. The medical ethics committee of Zhengzhou University People's Hospital approved the study program, and each patient signed an informed consent.

### 2.2 Research methods

#### 2.2.1 Measurement of FeNO and CANO

FeNO was measured by a hand‐held analyzer (Sunvou‐CA2122, Wuxi, China) according to the American Thoracic Society/European Respiratory Society (ATS/ERS) recommendations.^8^, ^9^ Before the test, the patient was introduced to the procedure in detail to ensure that the patient could complete the test independently in a quiet state. Subjects were informed to inhaled NO‐free air and exhaled via a mouthpiece at constant flow rates: 50 and 200 ml/s. Then, FeNO and CANO were automatically calculated by the analyzer.

#### 2.2.2 Pulmonary function test

After 30 min of FeNO and CANO measurement, pulmonary function test was performed (Spirolab III, Mill Thinking Medical Technology Co., Ltd.) according to the European Respiratory Society (ERS) recommendation.^10^ Several parameters were recorded in the form of percentage of predicted values containing forced vital capacity (FVC), forced expiratory volume in 1 s (FEV1), forced expiratory flow between 25 and 75 (FEF25–75), 75% forced expiratory flow, and 50% forced expiratory flow. In order to exclude the influence of the patient's height and weight, we took %pred. All subjects were asked to repeat the procedure at least thrice and the best values were noted.

Diagnostic criteria for small airway dysfunction are as follows: FEV1 > 80%pred, FVC > 80%pred, FEV1/FVC > 70%, and FEF25–75, FEF75, FEF50 < 65%pred at least two parameters. According to this criterion, the patients were divided into normal small airway function group and abnormal small airway function group.

### 2.3 Statistical analysis

SPSS software version 24.0 was used for data analysis. All continuous variables were checked for normal distribution by Kolmogorov–Smirnov normality test. Normally distributed variables are expressed as mean ± standard deviation, and skewed variables are expressed as the median (interquartile range [IQR]). The unpaired *T* test and *U* test were applied to examine the difference between the two groups for normally and nonnormally distributed parameters, respectively. The chi‐square test was used to compare categorical variables between two groups. Pearson's coefficients were utilized to assess the relationship between parameters. The receiver operating characteristic (ROC) curve was established to determine the value of CANO to predict small airway dysfunction. Statistical significance was considered to exist when *P* < 0.05.

## 3 RESULTS

### 3.1 Demographics of study subjects

A total of 144 patients were enrolled in this study, including 69 patients with normal small airway function and 75 patients with small airway dysfunction.

The characteristics of patients were shown in Table 1. There were no statistical differences in terms of age, sex, FVC%pred, FEV1%pred, and FeNO. CANO was significantly higher in the small airway dysfunction group compared with small airway normal group, while FEF25–75%pred, FEF50%pred, and FEF75%pred were significantly lower (*P* < 0.05).

**TABLE 1:** Comparison of general conditions and pulmonary function between normal and abnormal airway function groups

	Normal group (*n* = 69)	Abnormal group (*n* = 75)	*t*/*χ* ^2^	*P*
Sex (male/female)	31/38	35/40	0.044	0.834
Age (year)	44.55 ± 10.11	41.59 ± 9.88	1.779	0.077
CANO (ppb)	2.87 ± 1.50	7.28 ± 3.25	−10.607	<0.001
FVC%pred (%)	91.4 ± 5.9	91.3 ± 6.3	0.066	0.947
FEV1%pred (%)	92.2 ± 6.2	92.2 ± 6.1	0.078	0.944
FEF25–75%pred (%)	89.9 ± 7.5	55.0 ± 16.5	16.414	<0.001
FEF50%pred (%)	80.9 ± 6.8	46.4 ± 13.2	19.879	<0.001
FEF75%pred (%)	73.8 ± 5.0	41.9 ± 13.1	19.427	<0.001
FeNO (50 ml/s) (ppb)	21.88 ± 12.21	22.93 ± 10.63	−0.551	0.582
FeNO (200 ml/s) (ppb)	10.16 ± 5.01	10.55 ± 4.34	−0.627	0.532

### 3.2 The correlation between CANO and small airway function

**FIGURE 1:**
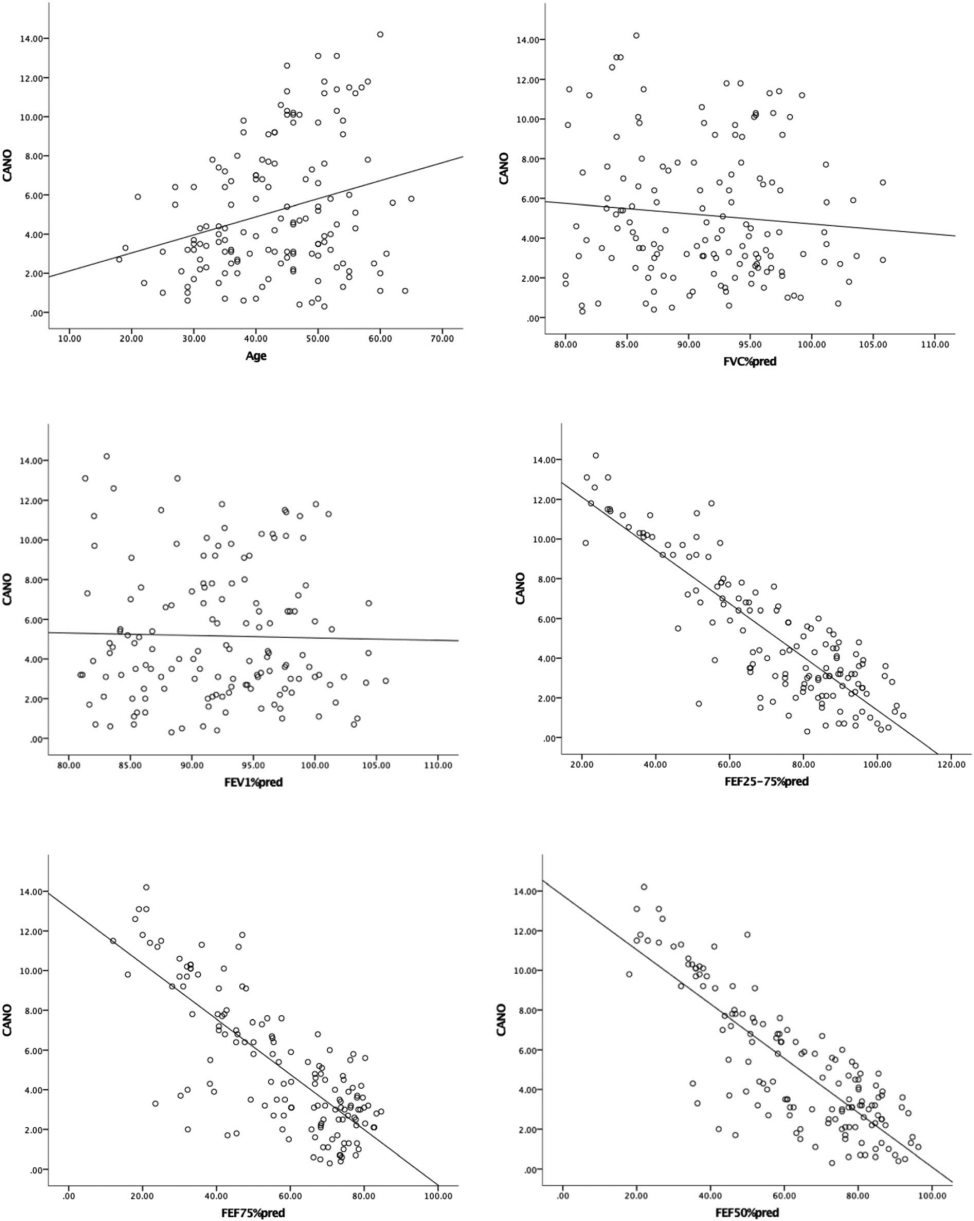
Correlation between CANO and various indicators

The application of Pearson correlations showed a positive correlation between CANO and age (*r* = 0.27, *P* = 0.001) and negative correlations between CANO, FEF25–75%pred, FEF50%pred, and FEF75%pred (*r* = −0.87, *P* < 0.001; *r* = −0.82, *P* < 0.001; *r* = −0.78, *P* < 0.001, respectively). There was no significant correlation between CANO, FVC%pred, and FEV1%pred (Table 2 and Figure 1).

**TABLE 2:** Correlation between CANO and various indicators

	Age	FVC%pred	FEV1%pred	FEF25–75%pred	FEF50%pred	FEF75%pred
*r*	0.27	−0.09	−0.02	−0.87	−0.82	−0.78
*P*	0.001	0.263	0.788	<0.001	<0.001	<0.001

### 3.3 Optimal CANO level to predict small airway dysfunction

**FIGURE 2:**
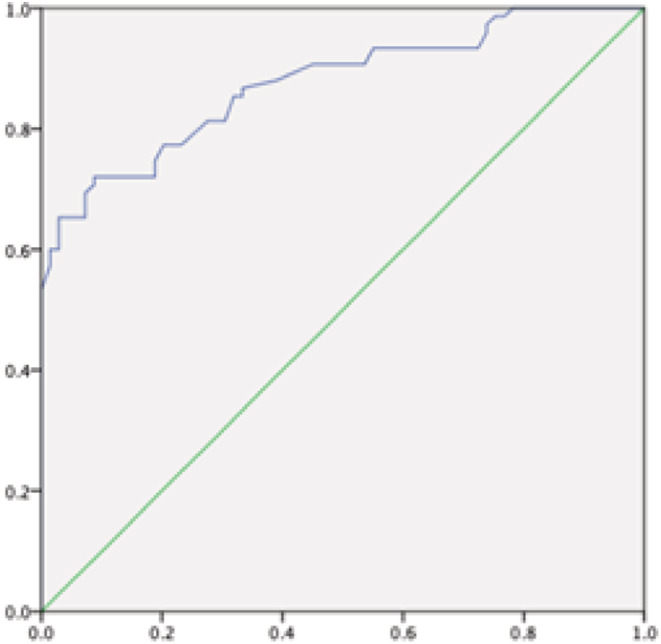
Receiver operating characteristic (ROC) curve of CANO in the diagnosis of small airway dysfunction in asthma patients

The ROC curve was performed to determine the best cutoff point. The area under the ROC curve (AUC) was 0.875. The sensitivity and specificity of CANO in predicting small airway dysfunction was 72% and 92% at a cutoff point of 5.3 ppb (Figure 2).

## 4 DISCUSSION

NO is produced by NO synthase (NOS), which catalyzes the production of l‐arginine and oxygen. Studies have shown that there are three main types of NOS in humans, including neuronal NOS (nNOS), inducible NOS (iNOS), and endothelial NOS (eNOS). These three kinks of NOS may be expressed in different cells of lung. The detection of FeNO at different flow rates provides more information for the inflammation in different parts of the airway and also facilitates the diagnosis and treatment of the disease. At present, this method is also being used in the evaluation and management of airway diseases. Among them, CANO was calculated using a two‐compartment model based on FeNO at different flow rates (50 and 200 ml/s). Our study indicates that CANO is highly correlated with indicators for evaluating small airway dysfunction in asthmatic patients, so CANO may be useful for the assessment of small airway dysfunction in asthmatic patients.

There were no statistically significant differences in gender and age between two groups. This provides the basis for comparison of other indicators. NO in orally exhaled air mainly originates from the respiratory epithelium but is also produced by activated macrophages and eosinophils. So it always elevates in patients with initial hormone‐dependent asthma and acute exacerbations.^11^ And FeNO may be normal in stable asthma. The alveolar concentration of NO is even an indirect marker of the inflammatory state of the distal portions of the lung.^12^ Our results showed no statistically significant difference in FeNO between the two groups of patients with stable asthma. However, some literatures have pointed out that FeNO can be used as an index to evaluate small airway dysfunction, and CANO is positively correlated with FeNO. Therefore, in future work, we need to collect more data to prove these ideas.

Our data show that CANO is negatively correlated with FEF25–75%pred, FEF50%pred, and FEF75%pred and has no significant correlation with FVC%pred and FEV1%pred but positively correlated with age. If FEV1, FVC, and FEV1/FVC are normal, an increase of CANO may indicate the change of small airway function. This is consistent with the published research results in the past. Whether there are differences in CANO in asthmatic patients has not been conclusive, suggesting that airway inflammation in asthmatic patients may involve different parts or have different subtypes.^13^, ^14^, ^15^, ^16^ A study of childhood asthma found that CANO was significantly elevated in symptomatic patients but not in asymptomatic and nonasthmatic patients. And CANO increased in patients with symptomatic but undiagnosed asthma and was negatively correlated with FEF50% and FEF75%, suggesting that NO in the alveoli may be associated with small airway dysfunction.^17^ Other studies have shown that patients with uncontrolled asthma have higher CANO than those with well‐controlled asthma.^18^ Berry et al. found that CANO in patients with refractory asthma was significantly higher than that in healthy and mild to moderate patients and was positively correlated with eosinophil count in alveolar lavage fluid, indicating that peripheral small airway and alveolar inflammation might play a role in refractory asthma.^19^ Studies have also found that CANO is significantly elevated in patients with asthma‐COPD overlap patients.^20^ And in chronic cough, it is also found that CANO is significantly elevated in cough variant asthma and nonasthmatic eosinophilic bronchitis.^21^ Sardón et al. found that the untreated group had higher CANO than the ICS treated group.^22^ In this study, we observed that CANO was negatively correlated with small airway function indicators (FEF25–75%pred, FEF50%pred, and FEF75%pred) and that CANO was of high value in the diagnosis of small airway dysfunction, suggesting that CANO could be used as a monitoring indicator for asthma management. In clinic, mild stable asthma patients may not show abnormal lung function, but peripheral small airway inflammation already exists, so inadequate treatment may even lead to changes in airway structure, thus aggravating the condition. Verschakelen pointed out that patients with asthma should consider the possibility of small airway injury before symptoms occur. In the course of drug reduction and step‐down therapy for patients in remission stage of asthma, ignoring the abnormality of small airway and terminating asthma treatment may lead to the recurrence of asthma.^23^ Therefore, the measurement of CANO levels and the assessment of lung function can help clinicians avoid underestimating the small airway dysfunction in asthma patients and tailor the appropriate treatment plan for asthmatic patients.

At present, it is still controversial whether the CANO is associated with small airway dysfunction in asthmatic patients. In one study of refractory asthma in children, CANO was correlated with FEF25–75%pred, whereas in another study of adult asthma, CANO was not correlated with FEF50%pred and FEF25–75%pred.^24^, ^25^ There have also been reported that CANO in patients with severe asthma is associated with peripheral small airway obstruction, which is assessed based by residual volume, functional residual volume, and closing volume. However, this correlation ceased to exist in patients with mild to moderate asthma.^26^ This difference may be related to the inclusion of patient characteristics, disease severity, and treatment modalities. In the future, we hope to include more cases to confirm the relationship between CANO and peripheral small airway dysfunction, so as to make the diagnosis simpler and easier to operate.

In this study, the AUC of CANO in the diagnosis of small airway dysfunction was 0.875, the optimal cutoff value was 5.3, the corresponding sensitivity was 72%, and specificity was 92%. The sensitivity is not very high. And more importantly, few studies on CANO as a diagnostic tool for small airway dysfunction have been reported so far. Moreover, the differences in the results of different studies may be related to the different inclusion and exclusion criteria. So we need better evidence.

In summary, in a group of patients with stable asthma, this study has shown a strong correlation between a test of small airways function (FEF25–75, FEF50, and FEF75) and CANO. The clinical value of this observation requires further investigation.

## CONFLICT OF INTEREST

There are no conflicts of interest, and its publication is supported by all the authors.

## AUTHOR CONTRIBUTIONS

Ke Wu, Jing Wang, and Limin Zhao conceived the study and reviewed the data. Jing Wang screened articles and wrote the manuscript. Xianliang Cheng, Xiangsong Chen, and Yanan Qi were responsible for data collection and reviewed the manuscript. All the authors revised and accepted the final version of the manuscript.

## Data Availability

Data sharing is not applicable to this article as no new data were created or analyzed in this study.
